# Association between Adiponectin and Leptin Receptor Genetic Polymorphisms and Clinical Manifestations of Metabolic Syndrome

**DOI:** 10.1155/2022/9881422

**Published:** 2022-09-08

**Authors:** Iuliana I. Shramko, Elizaveta S. Ageeva, Konstantin D. Maliy, Irina N. Repinskaya, Cyrill O. Tarimov, Iryna I. Fomochkina, Anatolii V. Kubishkin, Olga V. Ostapenko, Anna K. Gurtovaya, Suman Shekhar

**Affiliations:** S. I. Georgievsky Medical Academy of the Federal State Autonomous Educational Institution of Higher Education, V. I. Vernadsky Crimean Federal University, Simferopol 295000, Russia

## Abstract

Abdominal obesity coupled with polygenic hereditary defects is considered the initial event in the development of metabolic syndrome (MS). The purpose of this study was to analyse the frequency with which polymorphic loci of adiponectin (*ADIPOQ*) and leptin (*LEP*) genes occur in patients with MS and the association between the symptoms of MS and these polymorphisms. DNA was isolated from the whole blood of 207 patients with MS and 100 healthy individuals (control group) using the phenol-chloroform method. Gene polymorphisms were determined using real-time polymerase chain reaction (PCR). The most common variant of the *ADIPOQ* (rs2241766) gene among MS patients was the GT genotype. The A allele of the *LEP* (rs7799039) gene was found to be the most frequent in MS patients. The highest systolic blood pressure was found in carriers of the GG genotype of the *LEP* (rs7799039) gene. The carriers of the *ADIPOQ* (rs2241766) GT genotype were associated with the highest systolic blood pressure and *body mass index* (*BMI*); carriers of the *ADIPOQ* (rs2241766) GG genotype were associated with the highest diastolic blood pressure, hyperglycaemia, and elevated glycated haemoglobin (HbA1c). The results of this study allowed us to establish the unique gene variants associated with the risk of developing MS in the Crimean population.

## 1. Introduction

The current understanding of metabolic syndrome (MS) and diabetes mellitus type 2 (DM2) is that formation of their main components is influenced by the substrate-energy role played by lipocyte products and informational signalling molecules, such as leptin and adiponectin [1]. Leptin sensitivity is considered to be directly related to obesity and adipose tissue volume [2]. In obese mice, exposure of endothelial cells to leptin led to an increase in the level of reactive oxygen species. This increase was in turn associated with an increase in both Toll-like receptor of the second (TLR-2) and the forth type (TLR-4) expression and the components of their intracellular signalling pathways, as well as a decrease in the nicotinamide-adenine dinucleotide phosphate (*NADPH*) oxidase content in the intima [3]. The latter leads to a lack of expression and activity of Silent Information Regulator 1 proteins (SIRT1). Downregulation of SIRT1 leads to high acetylation of p53 and activation of the c-Jun N-terminal kinase (JNK)/activating protein-1 (AP-1) signalling pathway, which is also involved in inflammatory processes. In addition, the decrease in SIRT1 forces increased expression of peroxisome proliferator-activated receptor gamma (PPAR*γ*), which, through the stimulation of expression for a number of genes, causes an accumulation of lipids in adipocytes and obesity [4]. The results obtained by various research groups also demonstrate functional negative feedback: activation of TLR-4 in adipocytes initiates the increase of adiponectin synthesis, which in turn suppresses the effects of TLR-4. While adiponectin inhibits the TLR4 signalling pathway, leptin, on the contrary, enhances it [5–7]. Hence, in patients with MS, a high concentration of PPAR*γ* leads to the development of both leptin resistance and its corresponding effects on body weight, glucose level, lipid concentration, and other manifestations of MS.

A number of leptin gene mutations affecting its expression have been reported, one of the most significant of which was the single-nucleotide polymorphism -2549G<A (rs7799039) of the promoter region of *LEP* gene. Carriers of the AA genotype of *LEP* (rs7799039) gene had 2-fold higher serum leptin levels than carriers of the GA/GG genotypes [8]. The EPIC-Heidelberg study demonstrated that the genotype AA of the *LEP* (rs7799039) gene is associated with the development of obesity [9]. This polymorphism is located at the 5′ end of the promoter region of *LEP*, and it is suggested that this region may contain inhibitory elements from transcription in adipocytes [10]. The G allele of the *ADIPOQ* (rs2241766) gene is associated with the risk of hypertension and dyslipidaemia [11, 12]. The effect of this variant on pre-mRNA splicing or mRNA stability is well known, suggesting an allele-specific differential expression of adiponectin. The steady-state mRNA levels transcribed by the G allele were higher than those of the T allele in the adipose tissue of heterozygous subjects; multivariate linear regression analyses with age and gender adjusted showed that the dose of the G allele was associated with a reduction of approximately 1.12 kg/m^2^ in body mass index (BMI) [13]. Therefore, speculation that the gene *ADIPOQ* (rs2241766) polymorphism might be associated with obesity is reasonable. Consequently, the development of MS begins against the background of polygenic hereditary defects and an alimentary-hypodynamic lifestyle with abdominal obesity, which leads to a lipokine response as described above and contributes to the further development of the pathogenetic links of MS, in particular, confirming the prediagnostic effects of gene polymorphisms in diabetic patients. To select particular polymorphisms for our research, we used data from meta-analyses, which demonstrated a high degree of association of these single-nucleotide polymorphisms (SNP) and DM2. Thus, the gene *ADIPOQ* (rs*2*241766) polymorphism has been implicated in a susceptibility to DM2 in the Russian population [14, 15] as well as in European and Asian groups studied previously [16].

Single-nucleotide analysis carried out by Dagdan et al. [17] showed that gene *LEP* (rs7799039) polymorphism is most likely to be associated with an increased BMI or obesity as well as with a higher concentration of leptin. The gene *LEP* (rs7799039) polymorphism investigated by Bains et al. [18] and Alnory et al. [19] was associated with an increased risk of developing DM2 in Asian populations. The small numbers and varied populations in the published studies may partially account for the controversial results. Our study therefore is aimed at investigating the prevalence of the most significant leptin and adiponectin gene polymorphisms and their association with the pathogenesis of the primary clinical manifestations of MS.

## 2. Materials and Methods

### 2.1. Patient Selection

The study was performed using blood samples from 207 MS patients (107 women and 100 men) treated at Semashko Republican Hospital, Simferopol, and 100 community-based healthy volunteers (59 women and 41 men) as a control group (Table 1). All the examined individuals were Caucasians, in whose pedigrees there were no mixed marriages in at least three previous generations.

#### 2.1.1. Inclusion and Exclusion Criteria in the Study

*(1) (1) Inclusion Criteria for Patients*.

- Men or women aged ≥52.0 years but ≤70.0 years
- Patients with a verified diagnosis of MS arrived at using at least 3 conditions. The first and main condition was abdominal obesity (AO), accompanied by two additional conditions, according to the criteria developed by the IDF (*International Diabetes Federation*) in 2005 [20]: increased triglyceride (TG) levels greater than 1.7 mmol/L or TG-reducing therapy; high-density lipoprotein (HDL) levels less than 1.03 mmol/L for men and less than 1.29 mmol/L for women or specific cholesterol therapy; fasting hyperglycaemia with plasma glucose levels greater than 5.6 mmol/L or a previously established diagnosis of DM2; and/or hypertension (systolic arterial pressure ≥ 130 mmHg or diastolic arterial pressure ≥ 85 mmHg) or hypotensive therapy
- BMI of more than 30 kg/m^2^
- Willingness to participate voluntarily in the study and to sign an informed consent form

*(2) (2) Exclusion Criteria for Patients*.

- Men or women aged ≤52.0 years or ≥70.0 years
- Unstable DM2 (HbA1c target level > 7.0%, target fasting plasma glucose level > 7.0 mmol/L (2 hours after meals >9.0 mmol/L), and glomerular filtration rate according to the Chronic Kidney Disease Epidemiology Collaboration (CKD-EPI) of <80 mL/min/1.73 m^2^
- Chronic renal disease, heart failure, liver dysfunction, or malignant tumor
- Inability or unwillingness to participate in the study or to sign an informed consent form

*(3) (3) Inclusion Criteria for Control Subjects*.

- Healthy men or women aged ≥46.0 years but ≤62.0 years
- Individuals with a BMI of less than 30 kg/m^2^
- Willingness to participate voluntarily in the study and to sign an informed consent form

AO was assessed by waist measurement: men ≥ 94 cm, women ≥ 80 cm. BMI was calculated using the Quetelet formula and stratified according to the WHO classification [13]. Investigations were carried out following the rules of the Declaration of Helsinki of 1975, revised in 2013, and approved by the V.I. Vernadsky Crimean Federal University Ethics Committee (Protocol №. 8 from 17 January 2018). All subjects gave their informed consent for inclusion before they participated in the study.

### 2.2. Assays

Blood samples (10 mL) for analysis were drawn after a minimum of an 8 h overnight fast; serum was stored at -70°C until analysed. Fasting serum total cholesterol formation (mol/L) was studied using commercially available biochemical analyser kits, plasma glucose levels (mmol/L) were determined using an automatic glucometer, and HbA1c in whole blood (%) was determined using enhanced immunoturbidimetric test kits.

### 2.3. DNA Extraction and Genotyping

Genomic DNA was isolated from the whole blood of patients using the phenol-chloroform method; the A260/A280 ratio was assessed to determine the purity of the isolated DNA. Determination of single-nucleotide polymorphism of the markers studied (*LEP* (rs7799039) and *ADIPOQ* (rs2241766) genes [14, 15], Supplementary Table S1) was determined by real-time polymerase chain reaction (PCR) on a Bio-Rad CFX96 thermal cycler (USA) with real-time PCR kits, using the allelic discrimination method with hydrolysable fluorescent TaqMan probes. Homozygous variants of the genotype were established with a reproducible difference in threshold cycles of two or more (about 50% of all samples were examined two or more times to identify the results reliably).

The genotype study was carried out blindly, without information about phenotypic features, using a series of anonymous study numbers, followed by an independent analysis of the relationship between the results of the genotyping and the features of the clinical picture. Primers and fluorescent probes were synthesized by Syntol (Russia). The study was performed in the Center for Collective Use of Scientific Equipment Molecular Biology of the S. I. Georgievsky Medical Academy (structural division) of the V. I. Vernadsky CFU.

### 2.4. Statistical Analysis

Data were analysed using the Statistica 8.0 software package (StatSoft). Qualitative variables are described by absolute median (Me) and quartiles (Q1-Q3) and relative frequencies (percentages). The Mann–Whitney *U* test was used to assess the statistical significance of differences between patient groups. The critical level of significance was accepted at *p* < 0.05. The frequencies of allelic variants were determined using the Pearson method with the calculation of the *χ*^2^ value according to the formula as the ratio between the square of the difference between the observed and expected frequency to the expected frequency. The expected frequency of occurrence for the alleles was calculated (using the example of alleles A and B) based on the Hardy-Weinberg distribution law according to the formulas: expected frequency of allele A = (frequency of allele A)^2^ and expected frequency of allele B = (frequency of allele B)^2^. The expected frequency of the allele combination was calculated using the formula AB = 2 × (frequency of allele A) × (frequency of allele B). To compare the frequencies of allele combinations, the *χ*^2^ criterion was used with the Yates correction for continuity. The association of polymorphisms with DM2 was analysed by determining the odds ratio criterion (OR) and 95% confidence interval (95% CI). We used 207 patients with and 100 healthy individuals to increase the statistical power. Statistical power of the study was 0.8. That states 100 individuals enough to average accuracy research in the statistical power of 0.8 for general totality of 2.000.000 (total population of Crimea) (https://stattrek.com/survey-sampling/sample-size-calculator).

## 3. Results

In patients with MS the A allele of the *LEP* (rs7799039) gene occurred at a frequency of 48.1% (Table 2). Analyzing the frequencies of allelic combinations, the heterozygous combination of GA, found in 50.7% of patients, was most common. Homozygous variants of AA and GG were found in 22.7% and 26.6% of patients with MS, respectively.

Analysis of the frequency of the combinations of these allelic variants demonstrated that the *LEP* (rs7799039) gene heterozygous combination GA predominated among patients with MS and in healthy individuals (Table 2). The AA genotype was found with greater frequency in MS, while GG was found in the control group. The frequency of genotypes AA, AG, and GG of G(-2548)A of the *LEP* (rs7799039) gene polymorphism showed no differences in patients with MS compared with those in the control group (Table 2).

### 3.1. Polymorphism ADIPOQ (rs2241766) Gene

In patients with MS, the T allele of the *ADIPOQ* (rs2241766) gene was most common at 66.9% (Table 2). Analyses of the frequencies of the combinations of the *ADIPOQ* (rs2241766) gene allelic polymorphic variants among patients with MS revealed the homozygous combination of TT to be most common, with a frequency of 50.7%. In 32.4% of cases, a heterozygous variant of TG was discovered, and most rarely—in 16.9% of cases—a homozygous combination of GG was found (Table 2).

The frequencies of the *ADIPOQ* (rs2241766) gene genotypes TT, TG, and GG in MS patients and healthy individuals had statistically significant differences (Table 2). Analysis of the distribution of genotypes of this polymorphism revealed that the most common genotype among patients with MS was TT (50.7%), which exceeded the frequency of occurrence of carriers of this genotype in the control group (87.0%, *p* < 0.001). Among patients with MS, the genotypes TG and GG were statistically significantly different from those in the control group: TG (32.4%, *p* < 0.001, OR (95%*СI*) = 3.81 (1.79 − 8.09)) and GG (16.9%, *p* < 0.001, OR (95%*СI*) = 10.0 (2.25 − 44.7)).

**Figure 1:**
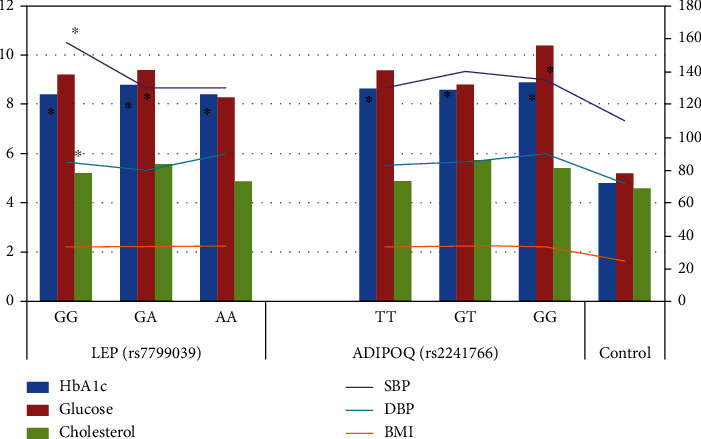
The clinical symptoms of MS for the *LEP* (rs7799039) and *ADIPOQ* (rs2241766) gene polymorphisms. HbA1c: glycated hemoglobin (%); glucose: fasting plasma glucose level (mmol/L); cholesterol: plasma level of cholesterol (mmol/L); SBP: systolic blood pressure (mmHg); DBP: diastolic blood pressure (mmHg); BMI: body mass index (kg/m^2^). ^∗^*p* < 0.05 MS vs. norm.

Clinical and laboratory evaluation of MS patients showed that the median index of HbA1c in patients with MS was 8.4% (range: 7.2-9.9%). The fasting blood glucose level in this group of patients was to 9.2 mmol/L (range: 6.1-11.1 mmol/L), and the cholesterol concentration was 5.1 mmol/L (range: 4.6-7.3 mmol/L). Median blood pressure indicators were systolic 130.0 mmHg (range: 110.0-140.0 mmHg) and diastolic 85.0 mmHg (range: 80.0-90.0 mmHg). The average BMI was calculated to be 33.9 kg/m^2^ (26.0-38.7 kg/m^2^). The control group demonstrated normoglycemia, normocholesterolemia, and physiological levels of HbA1c, as well as normotensia (Figure 1).

Analysis of the study data highlighted the lower cholesterol levels and glucose concentrations in carriers of the AA genotype of the *LEP* gene (rs7799039) compared with carriers of other variants of the genotype (Figure 1). The highest systolic blood pressure was found in carriers of the GG genotype of the *LEP* gene (rs7799039). The carriers of the *ADIPOQ* (rs2241766) gene's GT polymorphism demonstrated the highest systolic blood pressure, BMI, and cholesterol levels. Additionally, the GG genotype of the *ADIPOQ* (rs2241766) gene was associated with the highest numbers for diastolic blood pressure and hyperglycaemia, as well as the most significant values for HbA1c (Figure 1).

## 4. Discussion

Adiponectin and the genes of its receptors belong to the syntropic genes responsible for the development of visceral obesity and DM2 [21]. Carriers of single-nucleotide genetic polymorphisms, in particular *ADIPOQ* (rs2241766), may be predisposed to glucotoxicity, arterial hypertension, and hypercholesterolemia [22]. Clinical and genetic evaluation revealed that the development of visceral obesity in women was associated with carrying *ADIPOQ* (rs2241766) [23]. In the Crimean population, we found that GT was the most common genotype of the gene *ADIPOQ* (rs2241766) among patients with MS. In this genotype, we established a persistent association with arterial hypertension and with an increased BMI, which differs from the European and Asian data sets studied [22]. The genotype GG of the *ADIPOQ* (rs2241766) polymorphism in the Crimean population was associated with hyperglycemia and increased levels of glycated hemoglobin, which is also associated with the greatest risk of developing DM2, as has been shown in the Russian population [24]. In contrast to the data, which also demonstrated the relationship of this genotype with hyperglycemia and increased levels of glycated hemoglobin, in Crimea, the GG genotype was not associated with hypercholesterolemia (as in Asian populations) [25] or an increase in BMI (as in European and Russian populations) [24, 26]. We also established an association between this genotype and an increase in diastolic blood pressure, although European and Asian studies have not shown any relationship between the *ADIPOQ* (rs2241766) gene's GG genotype and an increase in systolic blood pressure [27].

Investigation of *LEP* (rs7799039) gene polymorphism revealed that the GA genotype was associated mostly with the development of MS, since it was associated with hyperglycemia and hypercholesterolemia. This association is unique to the Crimean population, despite the fact that European and Asian studies have established associations of DM and MS with other genotypes of *LEP* (rs7799039). At the same time, there are contradictory data concerning the effect of combined polymorphisms of the *ADIPOQ* and *LEP* genes. Thus, in the study of Yu et al. [28], polymorphisms in the *ADIPOQ* and *LEP* genes were not associated with a predisposition to obesity, while *ADIPOQ* G276T polymorphisms were associated with higher BMI. However, according to Zayani et al. [10], combined polymorphisms *ADIPOQ* 4522C<T and 276G<T contribute to obesity.

Few studies have been conducted on the polymorphism of the leptin and adiponectin receptor genes responsible for insulin resistance and the development of MS and DM2. Therefore, it seems reasonable to continue research in this direction with the subsequent stratification of genetic risk groups in relation to the development of MS and DM2.

## 5. Conclusions

Specificities in the frequencies of allelic variants of the *LEP* and *ADIPOQ* genes were found among MS patients in Crimea. The most common variant of the *ADIPOQ* (rs2241766) gene was GT. The A allele of the A/G promoter region of the *LEP* (rs7799039) gene was found to be the most frequent in MS patients. Certain polymorphic combinations related to MS pathogenesis were found only in the Crimean population. For instance, the highest systolic blood pressure was associated with the GG genotype of the *LEP* (rs7799039) gene, whereas the GT polymorphism of the *ADIPOQ* (rs2241766) gene was associated with the highest systolic blood pressure and BMI, and the GG genotype of the *ADIPOQ* (rs2241766) gene had the highest values for diastolic blood pressure, blood glucose levels, and HbA1c.

## Figures and Tables

**Table 1:** Characteristics of the whole study cohort.

	Control group		DM2 patients	
	Me	Q1-Q3	Me	Q1-Q3
Age (years)	58	46–62	61	52–70
HbA1c (%)	4.8	4.1-6.0	8.50	7.20-9.9
Fasting plasma glucose level (mmol/L)	5.2	3.6-5.8	9.2^∗^	6.1-11.1
Cholesterol (Mol/L)	4.6	3.6-6.2	5.1	4.6-7.3
BMI (kg/m^2^)	24.6	21.4-28.9	33.9^∗^	26.0-38.7
Systolic blood pressure (mmHg)	110.0	90.0-118.0	130.0^∗^	110.0-140.0
Diastolic blood pressure (mmHg)	72.0	65.0-80.0	85.0	80.0-90.0

Me: median; Q1-Q3: the first and third quartiles, ^∗^*p* value < 0.05 as compared to control.

**Table 2:** Allele and genotype frequency distribution of *LEP* (rs7799039) and *ADIPOQ* (rs2241766) gene polymorphisms in patients with MS.

*LEP* (rs7799039) gene				
Groups	MS patients, *n* (%)	Control group, *n* (%)	*χ* ^2^ *p*	

Alleles				
G	215 (51.9)	114.0 (57.0)	0.57	
А	199 (48.1)	86.0 (43.0)	0.57	
Allele combinations				
GG	55 (26.6)	31 (31.0)	0.64	
GA	105(50.7)	52 (52.0)	1.0	
AA	47 (22.7)	17 (17.0)	0.38	

*ADIPOQ (rs2241766) gene*				
Alleles	MS patients	Control group	*χ* ^2^ *p*	OR (95% СI)
	MS patients, *n* (%)	Control group, *n* (%)		

Alleles				
T	277 (66.9)	185 (92.5)	<0.001	0.17 (0.07-0.41)
G	137 (33.1)	15 (7.5)	<0.001	5.6 (2.4-13.0)
Allele combinations				
TT	105 (50.7)	87 (87.0)	<0.001	0.1 (0.07-0.31)
TG	67 (32.4)	11 (11.0)	<0.001	3.81 (1.79-8.09)
GG	35 (16.9)	2 (2.0)	<0.001	10.0 (2.25-44.7)

*n*: number of subjects; ns: not significant; OR: odds ratio.

## Data Availability

Nonidentifying data are available from the corresponding author upon reasonable request.

## Additional Points

*Limitations of the Study*. The factors limiting the significance of the results and conclusions of the study include the fact that the research was performed in a specific cohort of the population in the Republic of Crimea. Additionally, the inclusion criteria were strict, resulting in a relatively small cohort. Therefore, further analyses are required to enlarge the cohorts and clarify the SNP distribution characteristics.

## Supplementary Materials

Supplementary Materials Supplementary Table S1: sequences of primers and probes.
