# Supplementary material for: Association between Adiponectin and Leptin Receptor Genetic Polymorphisms and Clinical Manifestations of Metabolic Syndrome
Source: J Diabetes Res. 2022 Sep 8;2022:9881422. doi: 10.1155/2022/9881422 (PMC9477633; doi:10.1155/2022/9881422)
Supplement: Supplementary Materials — Supplementary Table S1: sequences of primers and probes. [file 9881422.f1.docx]

Table S1. Sequences of primers and probes

| Marker | Primer | Probe | Reference |
| --- | --- | --- | --- |
| *LEP* (rs7799039) gene | 5`CCTGTAATTTTCCCATGAGAAC  5`TGCAACATCTCAGCACTTAG | 5`FAM-CGTGCCCGACAGGGTTGCGCTGATCGGCACG-BHQ  5`R6G-CGTGCCCGACAGGGTTGCACTGATCGGCACG-BHQ | [14] |
| *ADIPOQ* (rs2241766) gene | 5`CAGGTAAGAATGTTTCTG 5`AGAGGAATCAGAATATGAA | 5`FAM-GAAGTAGACTCTGCTGAGATGG-BHQ  5`R6G-TATCAGTGTAGGAGGTCTGTGAGT-BHQ | [15] |
